# The Role of Mucin in the Toxicological Impact of Polystyrene Nanoparticles

**DOI:** 10.3390/ma11050724

**Published:** 2018-05-03

**Authors:** Iwona Inkielewicz-Stepniak, Lidia Tajber, Gavin Behan, Hongzhou Zhang, Marek W. Radomski, Carlos Medina, Maria J. Santos-Martinez

**Affiliations:** 1 Department of Medical Chemistry, Medical University of Gdansk, 80-211 Gdańsk, Poland; iwona.inkielewicz-stepniak@gumed.edu.pl; 2 School of Pharmacy and Pharmaceutical Sciences, Trinity College Dublin, the University of Dublin, Dublin 2, Ireland; ltajber@tcd.ie (L.T.); santosmm@tcd.ie (M.J.S.-M.); 3 Center for Research on Adaptive Nanostructures and Nanodevices, Trinity College Dublin, Dublin 2, Ireland; gbehan@tcd.ie (G.B.); hozhang@tcd.ie (H.Z.); 4 College of Medicine, University of Saskatchewan, Saskatoon, SK S7N 5E5, Canada; marek.radomski@usask.ca; 5 Trinity Biomedical Sciences Institute, Trinity College Dublin, the University of Dublin, Dublin 2, Ireland; 6 School of Medicine, Trinity College Dublin, the University of Dublin, Dublin 2, Ireland

**Keywords:** polystyrene, nanoparticles, mucin, DLS, QCM-D, cytotoxicity

## Abstract

The development of novel oral drug delivery systems is an expanding area of research and both new approaches for improving their efficacy and the investigation of their potential toxicological effect are crucial and should be performed in parallel. Polystyrene nanoparticles (NPs) have been used for the production of diagnostic and therapeutic nanosystems, are widely used in food packaging, and have also served as models for investigating NPs interactions with biological systems. The mucous gel layer that covers the epithelium of the gastrointestinal system is a complex barrier-exchange system that it is mainly constituted by mucin and it constitutes the first physical barrier encountered after ingestion. In this study, we aimed to investigate the effect of polystyrene NPs on mucin and its potential role during NP–cell interactions. For this purpose, we evaluated the interaction of polystyrene NPs with mucin in dispersion by dynamic light scattering and with a deposited layer of mucin using a quartz crystal microbalance with dissipation technology. Next, we measured cell viability and the apoptotic state of three enterocyte-like cell lines that differ in their ability to produce mucin, after their exposure to the NPs. Positive charged NPs showed the ability to strongly interact and aggregate mucin in our model. Positive NPs affected cell viability and induced apoptosis in all cell lines independently of their ability of produce mucin.

## 1. Introduction

Engineered nanoparticles (NPs) have been increasingly used in pharmaceuticals and industry for the past decade. Nanoparticles have been added to medicines, food, and cosmetics to improve their properties [1] and are being continuously developed to be used as drug delivery systems [2]. However, nano-sized counterparts frequently exhibit different properties from those found at the macro-scale. In fact, the small size of nanomaterials allow them to move through the body more freely than larger particles and their high surface area increases their reactivity [3]. Therefore, the effect of NPs on different biological systems has raised some concerns about their use in humans [3] and the potential toxicological effect of exposure to oral NPs is of increased interest and investigation [4].

The gut is a complex barrier-exchange system, and it is the most important route for macromolecules to enter the body. The epithelium of the small and large intestines is covered by a mucous gel layer which is in close proximity with the ingested materials including NPs. In fact, the gastrointestinal mucous, a complex network of highly branched glycoproteins and macromolecules, is the first barrier through which nutrients, oral drugs, and nanomaterials must interact with before entering the blood stream [5,6]. The transport of drug vectors across the gastrointestinal tract has significant implications for the development of novel oral drug delivery systems which could potentially have advantages over intravenous administration [7]. In this regard, numerous studies in the literature report the application of new strategies for improving the design of more efficient oral NP systems [8]. Nanotechnological tools have been also used in the entire food production chain, e.g., during cultivation (e.g., pesticides), industrial processing, or packaging [9]. The term ‘food additive’ plainly includes not only substances that are intentionally added to foods, but also substances that can migrate to food from packaging. Theoretical and experimental data have simultaneously demonstrated that the NP composites used in packaging had the potential and tendency to migrate to food [10]. Among those NPs, polystyrene are one of the most common NPs incorporated into different types of food packaging and they have been also used in cell imaging and drug delivery [11].

The main component of the gastrointestinal mucous is the glycoprotein mucin which has been found to reduce the diffusion of different drugs [12]. Indeed, mucins present the structural basis of defensive mucous gels to be found on human gastrointestinal surfaces. These gels provide surface entrapment and protection from toxic particles as well as from pathogens and chemical and physical insults whilst at the same time preserving appropriate properties for elimination by flow. It has been previously found that the transport of polystyrene NPs of increasing diameter in purified pig mucin is decreased with a cut-off at 500 nm [13]. Functionalized polystyrene NPs have been also previously used as models to explore the biological and toxicological effects of different NP surface properties on blood components such as monocytes, macrophages, and platelets and cells from different origins [14,15,16,17,18]. However, the contribution of the gastrointestinal mucous layer on NP cell toxicity has not been studied deeply.

The aim of the current study was to examine the interaction of monodisperse polystyrene NPs and gastrointestinal mucin and their potential toxicological effect on three enterocyte-like cell lines such as CaCo-2, HT-29, and LS174T. These three cell lines are commonly accepted model systems to study intestinal permeability, transepithelial transport, and interaction with drugs and food [19,20,21]. Caco-2 cells do not produce mucin unless previously stimulated [22] and HT-29 cells are able to produce a very limited amount of mucus [23]. However, mucin synthesis by the LS174T cell line has been well established [24].

## 2. Materials and Methods

### 2.1. Reagents

All reagents were purchased from Sigma-Aldrich (Dublin, Ireland) unless otherwise indicated.

### 2.2. Preparation and Characterization of Nanoparticles

Amine-modified, carboxyl-modified, and unmodified polystyrene NPs (~60 nm size) were purchased from Polysciences, Inc. (Edesheimer, Germany). The hydrodynamic size and zeta- potential values for all NPs tested were determined by dynamic light scattering (DLS) and laser Doppler velocimetry, respectively, using a Zetasizer Nano ZS (Malvern Instruments, Malvern, UK). Measurements were performed at 37 °C using a DTS 1060C clear disposable zeta potential cells and conducted in triplicate at a concentration of 100 µg/mL (with phosphate buffer solution-PBS). The morphology and size of the NPs were confirmed using a Carl Zeiss Orion Plus helium-ion microscope (HIM) (Carl Zeiss, Jena, Germany).

### 2.3. Interactions between Polystyrene NPs and Mucin Measured by Dynamic Light Scattering (DLS)

Dynamic light scattering was used to assess the degree of interactions of NPs with mucin using a method adapted from Chen et al. [25]. Firstly, 10 mg/L dispersions of porcine mucin in 30 mM NaCl solution at pH 5 and 7 were prepared and filtered through a 0.2245 μm PVDF membrane filter (Millipore, Cork, Ireland). The filtered mucin was aliquoted (as 10 mL portions) into 15 mL Corning centrifuge tubes (Cruinn, Dublin, Ireland) and equilibrated at 37 °C. Prior to the measurements, a tube containing 10 mL of the mucin dispersion was removed from the incubator and 10 μl of each of the NP type dispersions (supplied as 10% dispersions) was added to the tube and well mixed. DLS measurements sizing were carried out at 37 °C in ZEN0040 cuvettes using the Zetasizer Nano ZS at 0 h (immediately after mixing), 0.5, 1, 2, 4, 6, 24, and 72 h. Samples were kept at 37 °C between measurements and tightly capped while incubating to prevent water evaporation.

### 2.4. Interactions between Polystyrene NPs and a Mucin Layer under Flow Conditions by Quartz Crystal Microbalance with Dissipation (QCM-D)

Quartz crystal microbalance with dissipation (Q-Sense™ E4 system, Q-Sense AB, Gothenburg, Sweden) was used to investigate the interactions of NPs with a layer of mucin deposited on gold-coated quartz crystals as previously described [26]. The principle of analysis of QCM is based on the resonance frequency *f* of a quartz crystal induced by applying an alternating electric field across the crystal. Deposition of mass on the quartz surface decreases the crystal’s oscillation frequency *f* (negative f shift) and for thin, rigid, and uniformly distributed layers, *f* is proportional to the mass and it can be calculated using the Sauerbrey equation [27]. However, when a soft or thick layer is bound to the crystal, there is also a high dissipation *D* shift and in this case the mass can be underestimated by measuring only the *f.* Using a QCM-D, both parameters can be monitored simultaneously in real-time and mass and viscoelastic properties of the films can be characterized [28,29,30,31].

Prior to use, quartz gold crystals with a fundamental frequency of 4.95 MHz (QSX301, Particular Sciences, Dublin, Ireland) were cleaned in a 1:1:5 solution of 30% H_2_O_2_: 25% NH_3_: H_2_O at 70 °C, rinsed with ddH_2_O, dried with N_2_, and exposed to 10-min UV–ozone treatment (UV/Ozone ProCleaner, BioForce Nanotechnologies; Particular Sciences, Dublin, Ireland). Experiments were conducted at 37 °C and at a constant flow rate of 100 µL/min using a peristaltic pump (Ismatec, IMS 935; St. Neots, UK). Prior to depositing the mucin layer, a 30 mM NaCl solution (‘background solution’) was pumped through the flow cell containing the sensor to allow *f* and *D* readings to stabilize. Porcine mucin (25 mg/L) in background solution was then perfused and mucin allowed to deposit on the crystal surface until a plateau was achieved. Afterwards, background solution was perfused once again to remove any unbound or loosely bound mucin and afterwards the polystyrene NPs. Changes in *f* and *D* were recorded by QSoft401 software (v2.6, Q-Sense AB, Gothenburg, Sweden)) and the thickness of the mucin layer calculated applying the Voigt viscoelastic model by the QTools 3 software (Q-Sense AB, Gothenburg, Sweden) provided with the device as described previously by Wiecinski et al. [26].

### 2.5. Cell Culture

Intestinal epithelial cell lines, LS174T, HT-29, and Caco-2, were purchased from LGC standard (Teddington, UK). Cells were cultured in minimum essential medium (MEM) supplemented with 10% or 20% fetal bovine serum, 100 U/mL of penicillin, and 100 μg/mL of streptomycin. All cells were maintained at 37 °C in a humidified incubator containing 5% CO_2_. Cells were regularly split and subcultured up to ~80–90% confluence before experimental procedures.

LS174T, HT-29, and Caco-2 cell lines were treated with increasing concentrations (20, 50, and 100 µg/mL) of polystyrene NPs for 72 h. Working solutions were prepared in serum-free medium just before use.

### 2.6. Cytotoxicity

Membrane damage that results in LDH leakage is generally considered an irreversible cytotoxic event. Therefore, LDH leakage was used as a biomarker of cellular viability after exposure to polystyrene NPs. Lactate dehydrogenase (LDH) is an enzyme widely present in the cytosol that converts lactate to pyruvate. Therefore, if the plasma membrane integrity is disrupted, LDH leaks into the culture media. LDH-Cytotoxicity assay kit (BD Pharmingen^TM^, BD-Biosciences, Oxford, UK) was carried out according to the manufacturer’s protocol. Briefly, 1 × 10^4^ cells/well were seeded in 96-well plates and exposed to polystyrene NPs at various concentrations (20, 50, and 100 μg/mL). After 72 h of incubation, the 96-well plate was centrifuged at 2500 rpm for 5 min and 100 μL of supernatant and 100 μL of the reaction mix were mixed together in a fresh 96 well-plate and incubated for 30 min protected from the light. Following the addition of the stop solution, LDH activity was spectrophotometrically measured at λ: 492 nm using a microplate reader FLUOstar optima (BMG Labtech, Aylesbury, UK). LDH levels in the media versus the cells were quantified and compared to the control values according to the manufacturer’s instructions.

### 2.7. Flow Cytometry

The Annexin V-APC apoptosis detection kit (BD Pharmingen^TM^) was used to measure apoptosis/necrosis following the manufacturer’s instructions. Briefly, LS174T, HT-29, and Caco-2 cells (1 × 10^6^) were seeded into 25-cm^2^ tissue culture flasks. Cells were treated with various concentrations of polystyrene NPs. After 72 h of co-incubation, cancer cells were collected, washed twice with binding buffer, stained with 5 µL of Annexin V-APC and 5 µL of propidium iodide (PI) for 15 min in the dark, and analyzed by a BD FACSArray (BD Biosciences, Oxford, UK). Annexin V labeled with APC allowed to identify cells in early stage of apoptosis, and PI cells in medium and late stages of apoptosis/necrosis. A total of 10,000 events were acquired for each sample. The apoptotic rate was calculated as the percentage of Annexin V-positive and PI-negative cells divided by the total number of cells in the gated region.

### 2.8. Optical Microscopy

LS174T, HT-29, and Caco-2 at 80% confluence, were washed twice with basal media and incubated with increasing concentrations of polystyrene NPs for 72 h as described above. Untreated control cells (without NPs) were used as control. Cells were examined by an Olympus CKX41 inverted microscope (Olympus UK Ltd., Seashore, UK). Micrographs were taken by an Altra20 Soft Imaging System (Olympus; Mason Technology, Dublin, Ireland). 

### 2.9. Helium Ion Microscopy (HIM)

A Carl Zeiss Orion Plus helium-ion microscope was used to image the samples (NPs and Caco-2 cells in the presence/absence of polystyrene NPs). The biological samples cells were first fixed using 3% glutaraldehyde for 30 min at 37 °C and then dehydrated through ascending grades of ethanol (60% for 20 min, 80% for 20 min, 90% for 20 min, and finally 100% for 30 min repeated once). The working voltage was about 30 kV and the beam current was ~0.2 pA. The images were collected by using the Everhart–Thornley detector with a working distance of ~11 mm and line averaging of 255. The electron flood gun was used to mitigate sample charging.

### 2.10. Statistical Analysis

All statistical analyses were performed using GraphPad Prism v5 (GraphPad Software, San Diego, CA, USA.) and Origin v6.1 (OriginLab, Northampton, UK, MA, USA). Data is presented as mean ± SD of *n* ≥ 3. Statistical analysis of the mean difference between multiple groups was determined by one-way ANOVA followed by Tukey–Kramer multiple comparison post-test. A *p*-value < 0.05 was considered to be statistically significant.

## 3. Results

### 3.1. Nanoparticle Characterization

The charge of the NPs (positive for amine-modified and negative for carboxyl-modified and unmodified polystyrene NPs) was confirmed using a Zetasizer Nano. The median size for all polystyrene NPs tested are listed in Table 1. NPs were also examined by HIM and the micrographs showed spherical NPs with sizes in compliance with the manufacturer’s specifications.

### 3.2. Interactions between Polystyrene NPs and Mucin Measured by Dynamic Light Scattering (DLS)

Interactions of polystyrene NPs in a mucin dispersion at two different pH values (pH 5 and pH 7) were investigated by DLS. The size of mucin aggregates in a dispersion without NPs was around 150 nm indicative that mucin was forming clusters in the liquid. The hydrodynamic size of these clusters was not statistically significantly different for the different pHs (155 ± 5 nm at pH 5 and 147 ± 23 nm at pH 7).

**Figure 1:**
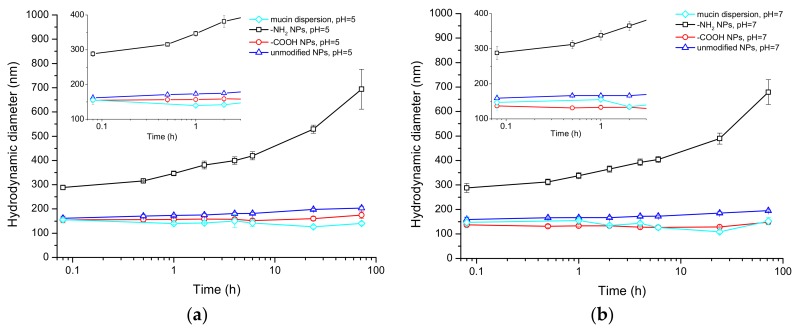
Hydrodynamic size of polystyrene NPs over time in mucin dispersion at (**a**) pH 5 and (**b**) pH 7 measured by Dynamic Light Scattering (DLS).

The addition of positively charged (-NH_2_ functionalized) NPs caused an immediate aggregation of mucin and the size of clusters increased dramatically to approximately 400 nm, as measured at the 2 h time point of the experiment, with a final size of several hundred nanometers at 72 h. On the other hand, the size of mucin clusters, when -COOH functionalized NPs were mixed with the mucin dispersion, was comparable to those of native aggregates over the time course of the study, while a slight increase (approximately 50 nm) in the size of the clusters was seen when the non-functionalized NPs were added. The pH of medium was a significant factor affecting the size of mucin clusters when -COOH functionalized NPs were studied, with the size of mucin agglomerates being smaller at pH 7 (Figure 1)

### 3.3. Interactions between Polystyrene NPs and Mucin under Flow Conditions Measured by Quartz Crystal Microbalance with Dissipation (QCM-D)

**Figure 2:**
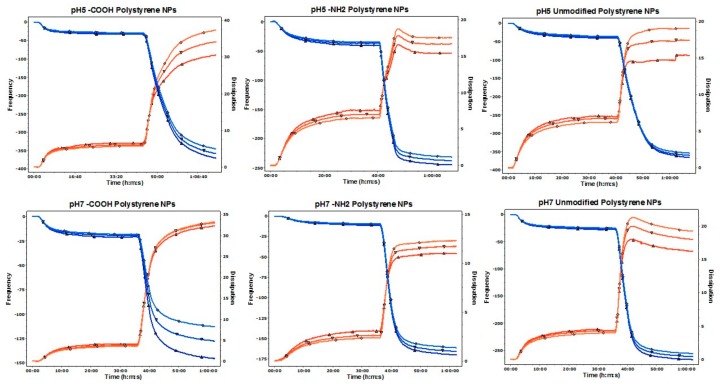
Representative traces for frequency *f* and energy dissipation *D* recorded by the QSoft sofware at three diferent overtones (fifth, seventh, and ninth). Frequency is represented as a blue line and its values shown in the left axis. Dissipation is represented as a red line and its values are shown in the right axis.

**Figure 3:**
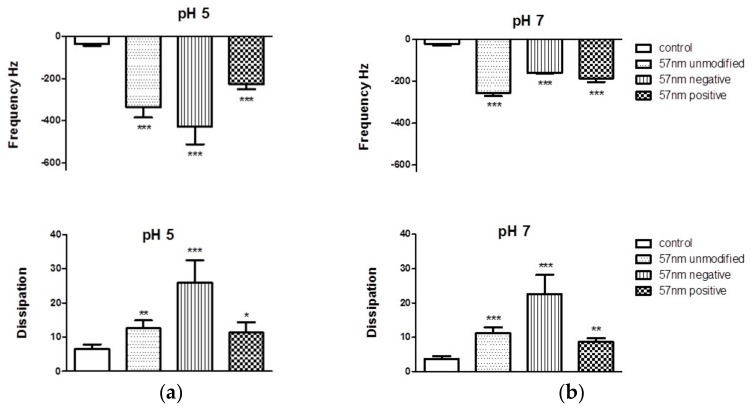
Statistical analysis of the interaction of polystyrene NPs with the mucin layer at (**a**) pH 5 and (**b**) pH 7 measured by quartz crystal microbalance with dissipation technology. Data are presented as mean ± SD (*n* = 3). One-way ANOVA followed by Tukey–Kramer multiple comparison post-test. * *p* < 0.05; ** *p* < 0.01 and *** *p* < 0.001 vs. control.

Interactions of the polystyrene NPs with a mucin layer deposited on a gold crystal at pH 5 and pH 7 were also investigated under flow conditions using a QCM-D. The perfusion of the mucin solution led to a decrease in the *f* and an increase in the *D* indicating the adsorption and deposition of a stable and reproducible layer of mucin. The perfusion of NPs led to a significant decrease in *f* and increase in the *D* when compared to the mucin layer before perfusion, indicating that all NPs tested interacted with the mucin layer. Changes in *D* induced by positively charged (-NH_2_ functionalized) NPs were less pronounced that the changes induced by negatively charged NPs. On the other hand, the most relevant changes in both *f* and *D* occurred after the perfusion of the negatively charged -COOH NPs at pH 5 (Figure 2 and Figure 3).

### 3.4. Positively Charged Polystyrene NPs Reduce Cell Viability

**Figure 4:**
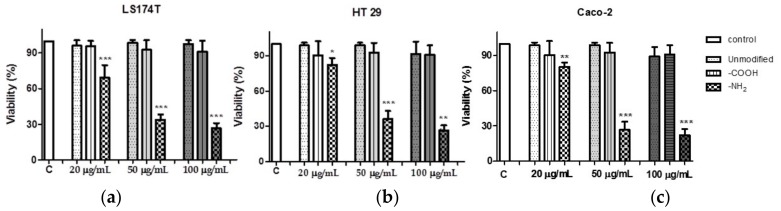
Effect of polystyrene nanoparticles on (**a**) LS174T; (**b**) HT-29; and (**c**) Caco-2 cell viability. Data are presented as mean ± SD of *n* ≥ 3. One-way ANOVA followed by Tukey–Kramer multiple comparison post-test. * *p* < 0.05; ** *p* < 0.01, and *** *p* < 0.001 vs. control.

**Figure 5:**
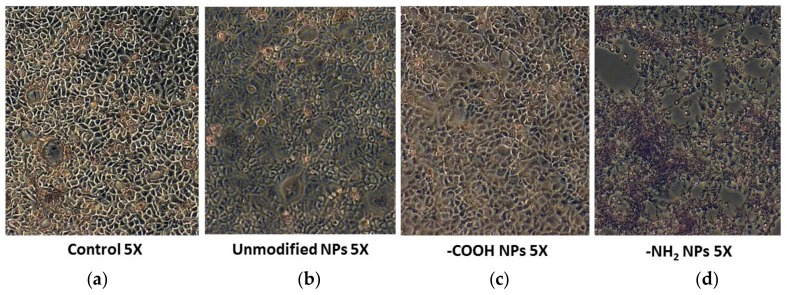
Effect of polystyrene nanoparticles on Caco-2 visualized by optical microscopy. Caco-2 cells in the (**a**) abscense and presence of (**b**) unmodified; (**c**) -COOH functionalized; and (**d**) -NH_2_ functionalized NPs showing the cytotoxic effect of the positively charge NPs.

**Figure 6:**
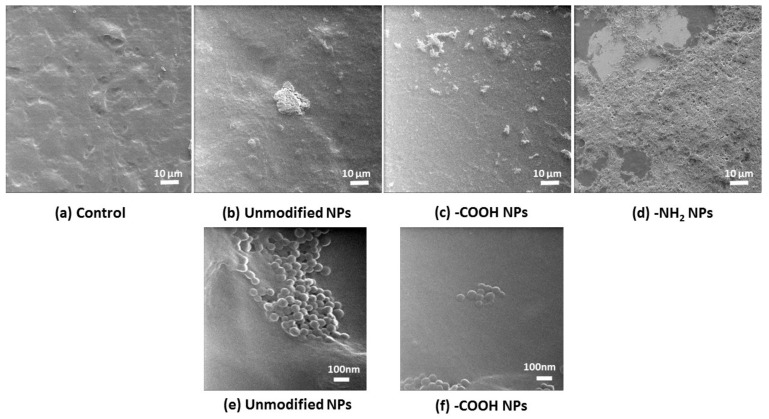
Effect of polystyrene nanoparticles on Caco-2 visualized by helium ion microscopy. Caco-2 cells in the abscense (**a**) and presence of (**b**) unmodified; (**c**) -COOH functionalized and (**d**) -NH_2_ functionalized NPs visualized by HIM showing the cytotoxic effect of the positively charge NPs. The lowest micrographs show the interaction of the (**e**) unmodified and (**f**) -COOH NPs with the cells at higher magnification and confirm the integrity of the cells.

The cytotoxic effect of increasing concentration of various polystyrene NPs on LS174T, HT29, and Caco2 cells was measure by the release of the cytosolic enzyme (LDH) to the media. A concentration dependent reduction in LS174T, HT29, and Caco2 and cell viability was found after incubation with -NH_2_ functionalized NPs. However, neither -COOH functionalized nor unmodified polystyrene NPs exerted any effect on cell viability (Figure 4). These results were corroborated by optical microscopy and HIM (Figure 5 and Figure 6).

### 3.5. Effect of Polystyrene NPs on Apoptosis

**Figure 7:**
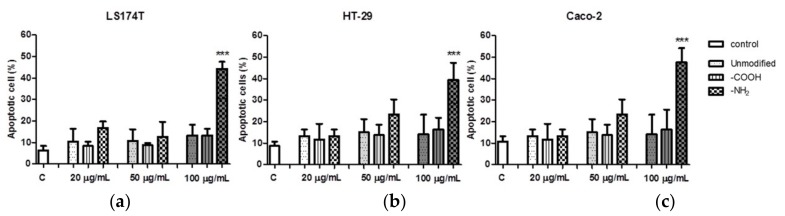
Effect of polystyrene nanoparticles on (**a**) LS174T; (**b**) HT-29; and (**c**) Caco-2 cell apoptosis. Data are presented as mean ± SD of *n* ≥ 3. One-way ANOVA followed by Tukey–Kramer multiple comparison post-test. *** *p* < 0.001 vs control.

We next studied polystyrene NPs-induced apoptosis by flow cytometry, since apoptosis has been recently identified as a major mechanism of cell death in exposure to nanomaterials [32]. Figure 7 shows that only positively charged (-NH_2_ functionalized) NPs at 100 ug/mL were able to induce apoptosis in all three cell lines studied. However, negatively charged (-COOH functionalized) and unmodified polystyrene NPs did not induce apoptosis when compared to control.

## 4. Discussion

The use of polystyrene NPs has increased in the last decade for many applications [33]. However, some concerns have raised with regards to their safety use in humans as they can be toxic to cells in various systems [34]. In this study, we aimed to investigate the interaction between different polystyrene NPs and mucin and whether or not mucin could be a protective factor against NP-induced cytotoxicity.

We have used two models using DLS and QCM-D, to investigate the interaction of polystyrene NPs with mucin. In the first model mucin in dispersion was incubated with NPs and changes in their size measured over time by DLS. In the second model, a reproducible and firmly bound layer of mucin was formed on the sensor surface, as demonstrated by the absence of changes in *f* or *D* after the perfusion of the background solution. Afterwards, NPs were perfused over the mucin layer and changes in *f* and *D* recorded. Prior to the studies, polystyrene NPs were characterized corroborating that their size and charge complied with the manufacturer’s specifications.

QCM and QCM-D have being previously used by other groups for investigating the muco-adhesive properties of biopolymers [35] and for looking at the interaction of PEGylated NPs with mucin [26]. More recently, QCM-D has been also applied for understanding the penetration (mucus permeability) of the NPs on the mucin layer based on the overtone response [36,37]. In fact, one of the main advantages that QCM-D offers vs. QCM is that the viscoelastic properties and changes of the layer deposited on the sensor can be analyzed looking at changes at the *D* factor.

Non-functionalized NPs have a significant number of -SO_3_ groups exposed on the surface resulting in a negative zeta potential value. The -COOH and -NH_2_ modified NPs had a negative and positive zeta potential value, respectively, as expected. The type of functional groups present on the surface of NPs plays a significant role in their interaction behavior with mucin. Electrostatic aggregation of mucin chains (containing negatively charged polysaccharide groups) by positively charged NPs was shown previously by Chen et al. [25] and corroborated in our experiments. Electrostatic cross-linking can lead to phase separation [38] which was also observed in this work as visual clouding of mucin dispersions containing positively charged NPs. Reduced mucin swelling has been previously reported [25] and attributed to changing mucin’s rheological properties. In fact, -NH_2_ NPs penetrated and compacted the mucin layer as demonstrated by the changes observed in all the overtones in the *D* factor as previously described by Borros et al. group [36,37]. The -COOH functionalized NPs studied in this work did not induce mucin aggregation as measured by DLS, which is consistent with the previously described electrostatic repulsion between the NPs and mucin chains. However, it has been previously reported that although -COOH polystyrene NPs do not appear to aggregate heavily in mucus, they may still adhere as single particles to the mucus network (Dawson, Krauland et al. 2004). In our studies using QCM-D, we have demonstrated that both unmodified and -COOH NPs are able to interact with a mucin layer as both of them led to a decrease in *f* (increase in mass) and an increase in *D* (viscoelasticity of the mucin layer). In fact, while charge undoubtedly plays a key role triggering—or not triggering—extensive mucin aggregation, the composition of the surface is also of importance, as clearly demonstrated for the different behavior of the -SO_3_ and -COOH NPs in both models.

Mucin undergoes significant conformational changes that are pH-driven, from a random coil conformation at neutral pH to extended random coils at lower pH [39]. A decrease in environmental pH leads to the breakage of electrostatic interactions, a greater exposure of hydrophobic regions of the mucin chain and thus may result in a slightly different type and magnitude of interactions with NPs. This is especially visible for -COOH NPs that generated mucin clusters of different sizes in media with pH 5 and pH 7 and also to significant variations in *f* when interacting with the mucin layer. However, -SO_3_ functionalized NPs caused a slight—but still significant—aggregation of mucin chains and a significant interaction with the mucin layer in terms of both *f* and *D*, which, considering the strong negative surface charge of these NPs, is surprising. It can be hypothesized that these NPs may cause disruption of electrostatic interactions and H-bonds due to competition between the strongly charged moieties of NPs and mucin, leading to the destabilization of the mucin structure especially in dispersion. Such structural changes can lead to entrapment of NPs and therefore, to an increase in the size of mucin aggregates.

The mucosal surfaces of the gastrointestinal tract are the first site where foods and drug delivery systems encounter the host. Mucin glycoproteins secreted by mucous producing cells in the epithelium produce a layer of viscous mucous which acts as a protective barrier between the underlying epithelium and the lumen containing noxious agents, destructive hydrolases, and microorganisms [40]. The human intestinal epithelium is made up of two major cell phenotypes which are enterocytes that transport nutrients and goblet cells that make store and secrete the mucin glycoproteins. We selected three intestinal epithelial cell lines that differ in their ability to produce mucin to study the effect of polystyrene NPs on cell viability and apoptosis. The Caco-2 cell line forms polarized monolayers in culture and differentiates into cells with high homology to enterocytes in the intestinal epithelium [41]. The HT-29 cultures are heterogeneous and in the post-confluent state consist of >95% undifferentiated cells and a small proportion of differentiated mucin-secreting and absorptive cells [42]. However, LS174T cell line has high levels of mucin [24]. Indeed, LS174T cells exhibit numerous and prominent mucinous secretory granules with variable electron densities [43].

Co-incubation of polystyrene NPs with the cell lines led to a concentration dependent reduction in Caco2, HT29, and LS174T cell viability after exposure to -NH_2_ polystyrene NPs, but not with unmodified and -COOH polystyrene NPs. These results were corroborated by optical microscopy and HIM where the morphology of the cells exposed to positive nanoparticles appeared completely distorted. Obviously, the size, shape, and charge of NPs are important factors contributing to their deleterious effects on cells. Several works have reported cell death activated by cationic NPs in various cell types. It has been previously found that positively charged polystyrene NPs—in contrast to negatively charged NPs—cause mitochondrial damage, ATP depletion, and cell death in human bronchial epithelial cells [14,44]. It has also been reported that positively charged silicon NPs exerted higher toxicity towards the Caco-2 cells compared to neutral and negatively charged NPs [45]. Therefore, we next studied whether or not polystyrene NPs could cause cell death via apoptosis. Increase in intracellular reactive oxygen species and consequent damage to the mitochondria have been regarded as crucial steps in the toxicity induced by positive NPs, which often lead to cell death via apoptotic mechanisms [44]. We found that our positively charged NPs were able to induce apoptosis as measured by flow cytometry in all cell lines tested. Our results are in agreement with previous studies where amine-modified polystyrene NPs mediated cell death through apoptotic mechanisms involving caspase-3-, 7-, and 9-mediated cytotoxicity in an astrocytoma cell line [46]. Therefore, taken together, our results suggest that positively charged polystyrene NPs exert toxicological effects equally on mucin- and no mucin-secreting intestinal epithelial cells. Further studies are guaranteed to provide more in depth information of the toxicity of positively charged NPs on intestinal epithelial cells including the extent of NPs internalization though the mucus barrier, kinetics of NPs uptake, and NP uptake mechanisms.

## Figures and Tables

**Table 1:** Size of polystyrene nanoparticles

Polystyrene Nanoparticles	Size (nm)/PdI ^1^
Amine-modified (-NH_2_)	56.93 ± 0.37/0.055 ± 0.007
Carboxyl-modified (-COOH)	61.35 ± 0.59/0.062 ± 0.008
Unmodified	63.93 ± 0.48/0.021 ± 0.006

^1^ PdI: Polydispersity index.

## References

[1] Tiede K., Boxall A.B.A., Tear S.P., Lewis J., David H., Hassellöv M. (2008). Detection and characterization of engineered nanoparticles in food and the environment. Food Addit. Contam. Part A.

[2] Faraji A.H., Wipf P. (2009). Nanoparticles in cellular drug delivery. Bioorg. Med. Chem..

[3] Seaton A., Donaldson K. (2005). Nanoscience, nanotoxicology, and the need to think small. Lancet.

[4] Kessler R. (2011). Engineered Nanoparticles in Consumer Products: Understanding a New Ingredient. Environ. Health Perspect..

[5] Cone R.A. (2009). Barrier properties of mucus. Adv. Drug Deliv. Rev..

[6] Lai S.K., Wang Y.-Y., Wirtz D., Hanes J. (2009). Micro- and macrorheology of mucus. Adv. Drug Deliv. Rev..

[7] Win K.Y., Feng S.-S. (2005). Effects of particle size and surface coating on cellular uptake of polymeric nanoparticles for oral delivery of anticancer drugs. Biomaterials.

[8] Date A.A., Hanes J., Ensign L.M. (2016). Nanoparticles for oral delivery: Design, evaluation and state-of-the-art. J. Control. Release.

[9] Rashidi L., Khosravi-Darani K. (2011). The Applications of Nanotechnology in Food Industry. Crit. Rev. Food Sci. Nutr..

[10] Loyo-Rosales J.E., Rosales-Rivera G.C., Lynch A.M., Rice C.P., Torrents A. (2004). Migration of Nonylphenol from Plastic Containers to Water and a Milk Surrogate. J. Agric. Food Chem..

[11] Wang T., Wang L., Li X., Hu X., Han Y., Luo Y., Wang Z., Li Q., Aldalbahi A., Wang L. (2017). Size-Dependent Regulation of Intracellular Trafficking of Polystyrene Nanoparticle-Based Drug-Delivery Systems. ACS Appl. Mater. Interfaces.

[12] Norris D.A., Puri N., Sinko P.J. (1998). The effect of physical barriers and properties on the oral absorption of particulates. Adv. Drug Deliv. Rev..

[13] Norris D.A., Sinko P.J. (1997). Effect of size, surface charge, and hydrophobicity on the translocation of polystyrene microspheres through gastrointestinal mucin. J. Appl. Polym. Sci..

[14] Chiu H.-W., Xia T., Lee Y.-H., Chen C.-W., Tsai J.-C., Wang Y.-J. (2015). Cationic polystyrene nanospheres induce autophagic cell death through the induction of endoplasmic reticulum stress. Nanoscale.

[15] Loos C., Syrovets T., Musyanovych A., Mailänder V., Landfester K., Nienhaus G.U., Simmet T. (2014). Functionalized polystyrene nanoparticles as a platform for studying bio-nano interactions. Beilstein J. Nanotechnol..

[16] Santos-Martinez M.J., Inkielewicz-Stepniak I., Medina C., Rahme K., D’Arcy D.M., Fox D., Holmes J.D., Zhang H., Radomski M.W. (2012). The use of quartz crystal microbalance with dissipation (QCM-D) for studying nanoparticle-induced platelet aggregation. Int. J. Nanomed..

[17] Santos-Martinez M.J., Tomaszewski K.A., Medina C., Bazou D., Gilmer J.F., Radomski M.W. (2015). Pharmacological characterization of nanoparticle-induced platelet microaggregation using quartz crystal microbalance with dissipation: Comparison with light aggregometry. Int. J. Nanomed..

[18] Smyth E., Solomon A., Vydyanath A., Luther P.K., Pitchford S., Tetley T.D., Emerson M. (2015). Induction and enhancement of platelet aggregation in vitro and in vivo by model polystyrene nanoparticles. Nanotoxicology.

[19] Calcagno A.M., Fostel J.M., Orchekowski R.P., Alston J.T., Mattes W.B., Siahaan T.J., Ware J.A. (2005). Modulation of Cell Adhesion Molecules in Various Epithelial Cell Lines after Treatment with PP2. Mol. Pharm..

[20] Resta-Lenert S., Das S., Batra S.K., Ho S.B. (2011). Muc17 protects intestinal epithelial cells from enteroinvasive E. coli infection by promoting epithelial barrier integrity. Am. J. Physiol. Gastrointest. Liver Physiol..

[21] Wood K.M., Stone G.M., Peppas N.A. (2010). The effect of complexation hydrogels on insulin transport in intestinal epithelial cell models. Acta Biomater..

[22] Navabi N., McGuckin M.A., Lindén S.K. (2013). Gastrointestinal Cell Lines Form Polarized Epithelia with an Adherent Mucus Layer when Cultured in Semi-Wet Interfaces with Mechanical Stimulation. PLoS ONE.

[23] Gagnon M., Zihler Berner A., Chervet N., Chassard C., Lacroix C. (2013). Comparison of the Caco-2, HT-29 and the mucus-secreting HT29-MTX intestinal cell models to investigate Salmonella adhesion and invasion. J. Microbiol. Methods.

[24] Byrd J.C., Nardelli J., Siddiqui B., Kim Y.S. (1988). Isolation and Characterization of Colon Cancer Mucin from Xenografts of LS174T Cells. Cancer Res..

[25] Chen E.Y.T., Wang Y.-C., Chen C.-S., Chin W.-C. (2010). Functionalized Positive Nanoparticles Reduce Mucin Swelling and Dispersion. PLoS ONE.

[26] Wiecinski P.N., Metz K.M., Mangham A.N., Jacobson K.H., Hamers R.J., Pedersen J.A. (2009). Gastrointestinal biodurability of engineered nanoparticles: Development of an in vitro assay. Nanotoxicology.

[27] Sauerbrey G. (1959). Verwendung von Schwingquarzen zur Wägung dünner Schichten und zur Mikrowägung. Z. Phys..

[28] Dixon M.C. (2008). Quartz Crystal Microbalance with Dissipation Monitoring: Enabling Real-Time Characterization of Biological Materials and Their Interactions. J. Biomol. Tech. JBT.

[29] Marx K.A. (2003). Quartz Crystal Microbalance: A Useful Tool for Studying Thin Polymer Films and Complex Biomolecular Systems at the Solution—Surface Interface. Biomacromolecules.

[30] Fredriksson C., Kihlman S., Rodahl M., Kasemo B. (1998). The Piezoelectric Quartz Crystal Mass and Dissipation Sensor: A Means of Studying Cell Adhesion. Langmuir.

[31] Höök F., Rodahl M., Brzezinski P., Kasemo B. (1998). Energy Dissipation Kinetics for Protein and Antibody−Antigen Adsorption under Shear Oscillation on a Quartz Crystal Microbalance. Langmuir.

[32] Hsin Y.-H., Chen C.-F., Huang S., Shih T.-S., Lai P.-S., Chueh P.J. (2008). The apoptotic effect of nanosilver is mediated by a ROS- and JNK-dependent mechanism involving the mitochondrial pathway in NIH3T3 cells. Toxicol. Lett..

[33] Youssef A.M., Kamel S., El-Samahy M.A. (2013). Morphological and antibacterial properties of modified paper by PS nanocomposites for packaging applications. Carbohydr. Polym..

[34] Wang F., Bexiga M.G., Anguissola S., Boya P., Simpson J.C., Salvati A., Dawson K.A. (2013). Time resolved study of cell death mechanisms induced by amine-modified polystyrene nanoparticles. Nanoscale.

[35] Chayed S., Winnik F.M. (2007). In vitro evaluation of the mucoadhesive properties of polysaccharide-based nanoparticulate oral drug delivery systems. Eur. J. Pharm. Biopharm..

[36] Oh S., Borrós S. (2016). Mucoadhesion vs. mucus permeability of thiolated chitosan polymers and their resulting nanoparticles using a quartz crystal microbalance with dissipation (QCM-D). Colloids Surf. B Biointerfaces.

[37] Oh S., Wilcox M., Pearson J.P., Borrós S. (2015). Optimal design for studying mucoadhesive polymers interaction with gastric mucin using a quartz crystal microbalance with dissipation (QCM-D): Comparison of two different mucin origins. Eur. J. Pharm. Biopharm..

[38] Umerska A., Paluch K.J., Inkielewicz-Stępniak I., Santos-Martinez M.J., Corrigan O.I., Medina C., Tajber L. (2012). Exploring the assembly process and properties of novel crosslinker-free hyaluronate-based polyelectrolyte complex nanocarriers. Int. J. Pharm..

[39] Bansil R., Turner B.S. (2018). The biology of mucus: Composition, synthesis and organization. Adv. Drug Deliv. Rev..

[40] Atuma C., Strugala V., Allen A., Holm L. (2001). The adherent gastrointestinal mucus gel layer: Thickness and physical state in vivo. Am. J. Physiol. Gastrointest. Liver Physiol..

[41] Rousset M. (1986). The human colon carcinoma cell lines HT-29 and Caco-2: Two in vitro models for the study of intestinal differentiation. Biochimie.

[42] Huet G., Kim I., de Bolos C., Lo-Guidice J.M., Moreau O., Hemon B., Richet C., Delannoy P., Real F.X., Degand P. (1995). Characterization of mucins and proteoglycans synthesized by a mucin-secreting HT-29 cell subpopulation. J. Cell Sci..

[43] Bu X.-D., Li N., Tian X.-Q., Huang P.-L. (2011). Caco-2 and LS174T cell lines provide different models for studying mucin expression in colon cancer. Tissue Cell.

[44] Xia T., Kovochich M., Liong M., Zink J.I., Nel A.E. (2008). Cationic Polystyrene Nanosphere Toxicity Depends on Cell-Specific Endocytic and Mitochondrial Injury Pathways. ACS Nano.

[45] Bhattacharjee S., de Haan L.H.J., Evers N.M., Jiang X., Marcelis A.T.M., Zuilhof H., Rietjens I.M.C.M., Alink G.M. (2010). Role of surface charge and oxidative stress in cytotoxicity of organic monolayer-coated silicon nanoparticles towards macrophage NR8383 cells. Part. Fibre Toxicol..

[46] Bexiga M.G., Varela J.A., Wang F., Fenaroli F., Salvati A., Lynch I., Simpson J.C., Dawson K.A. (2011). Cationic nanoparticles induce caspase 3-, 7- and 9-mediated cytotoxicity in a human astrocytoma cell line. Nanotoxicology.

